# Production of an Esterquat-Based Novel Softening Agent and Its Impact on Leather and Textile Quality

**DOI:** 10.1021/acsomega.4c09502

**Published:** 2025-03-03

**Authors:** Ali Yorgancioglu, Ersin Onem, Saltanat Sabyrkhanova

**Affiliations:** a Department of Leather Engineering, Faculty of Engineering, Ege University, Bornova-Izmir 35100, Turkey; b Department of Technology and Design of Textile Materials, Higher School of Light and Food Engineering, South Kazakhstan University named after M. Auezov, Tauke Khan Avenue, Shymkent 160012, Kazakhstan

## Abstract

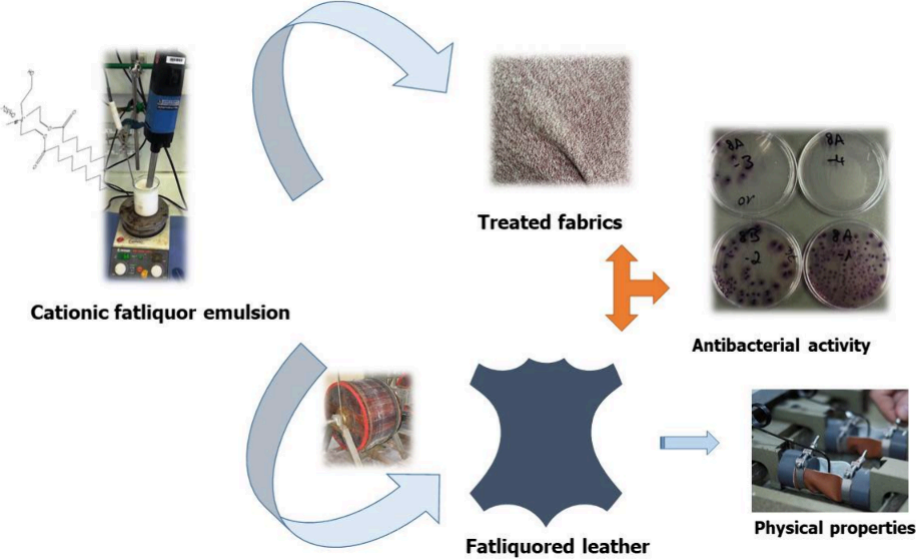

This study focuses on the development of an esterquat-based cationic softening emulsion for use in leather and textile applications. Esterquats, known for their excellent biodegradability and softening properties, were formulated into oil-in-water emulsions and applied to both chrome-tanned leather and cotton-polyester textiles. The stability of the emulsion was confirmed through particle size and zeta potential analyses. The emulsion was applied to chrome-tanned leather at concentrations ranging from 0.5 to 4%, followed by assessments of tensile strength, tear resistance, and water repellence. Additionally, the emulsion’s effect on dye uptake was evaluated using UV-spectrophotometer analysis, with results showing up to 88.98% dyestuff exhaustion at 4% emulsion concentration. Antibacterial efficacy against *Escherichia coli* and *Staphylococcus aureus* was also tested, revealing reductions up to 57.83 and 79.46%, respectively. For textile applications, the emulsion was applied to cotton and polyester fabrics via the exhaust method, followed by drying and curing. Softening power was assessed using a panel feel test with significant improvements observed, particularly on cotton fabrics. These findings suggest that esterquat-based emulsions offer a promising, eco-friendly alternative for improving the physical and functional properties of leather and textiles.

## Introduction

The textile and leather industries, known for their environmental pollution and ongoing challenges with meeting ecological criteria, must prioritize adopting sustainable production practices.^1^ Ensuring the transfer of production to future generations demands the use of environmentally friendly techniques and technologies as well as incorporating developments in chemical technology into manufacturing processes.

In textile and leather production, high volumes of water and a wide variety of chemicals with different properties are used. Especially in the leather industry, large amounts of solid and liquid wastes generated during production cause harmful effects on both human health and the environment.^2^ Due to this situation, the leather industry is seen as a sector that pollutes the environment despite its economic return. Therefore, reducing the use of chemicals and the pollution load in leather production is a new clean production approach that should be emphasized ecologically and economically.

Despite efforts to develop more environmentally friendly technologies in leather production, no truly innovative, practical, and sustainable solutions have been successfully implemented. As a result, the environmental challenges facing the leather industry continue to escalate. Especially in recent years, the leather industry has adopted the goals of reducing pollution, simplifying production processes, and ensuring sustainable development. However, there are significant challenges in current leather processing methods that make it difficult to achieve these goals, including low chemical absorption rates, the generation of large quantities of contaminated waste, low biodegradability of the chemicals used, and complex production processes.^3^

The fatliquoring process is one of the most critical stages in leather production, performed alongside retanning and dyeing as one of the final steps in the process. During fatliquoring, a thin film of oil is formed on the fibers, allowing them to slide smoothly over each other without friction. This mechanism imparts the finished leather with softness, elasticity, and flexibility, which are essential for its functional and aesthetic properties.^4^

The mechanism of fatliquoring relies on the homogeneous penetration of oil emulsions between leather fibers, their ability to form stable bonds with the fibers, and the effective lubrication of the fibers by coating their surfaces. These emulsions replace the natural oils lost during degreasing, preventing the fibers from drying out or sticking together, while also providing insulation between the fibers.^5^

The interaction and compatibility among the leather, oil, and emulsifying agents play a vital role in achieving the desired effects. The fatliquoring emulsion should soften the leather while maintaining its structure without causing excessive stretching.^6^ In addition to enhancing softness and elasticity, fatliquoring emulsions contribute to improving other critical properties of leather, including fullness, tensile strength, tear resistance, elongation, light and heat fastness, water resistance, and aging resistance. This multifunctional role underscores the importance of the fatliquoring process in determining the quality of the final leather product.^7^

Although most of the fatliquors used in the industry are anionic surfactants, cationic surfactants are also used to create a final touch on the leather.^8^ Cationic fatliquoring agents typically contain quaternary ammonium groups, which confer distinctive properties to leather, such as enhanced softness, antistatic effects, and antibacterial functionality. These agents can be applied during the pretanning process to promote better penetration and distribution of chrome tanning agents. Additionally, they are useful in secondary fatliquoring, where they help to fix anionic retanning agents, fatliquors, and dyes, while also fine-tuning the leather’s texture and feel.^9^ However, in traditional leather production, especially in chrome-tanned leathers, anionic surfactants are generally used after neutralization, and cationic surfactants are not preferred in the neutralization pH range. For this reason, the use of cationic fatliquoring emulsions is very limited.

In the globally changing market of the leather industry, there is a need to develop sophisticated, ecological, residue-free fatliquor emulsions rather than conventional fatliquors for economical and environmentally friendly production. Recent advancements in green chemistry have opened avenues for more sustainable practices in leather processing. One such development is the use of cationic fatliquoring emulsions, which have shown potential in improving the environmental footprint of leather manufacturing.^10,11^ These emulsions, especially those containing esterquats, offer an eco-friendly approach to leather treatment, aligning with the industry’s shift toward cleaner production technologies.^9^

Esterquat is one of the varieties of cationic emulsions in the textile industry due to its unique properties and benefits. The term “esterquat” was first introduced in 1931 in a patent application to describe a specific quaternary ammonium salt containing an ester linkage within its cation.^12^ They are quaternary ammonium compounds that contain two long fatty acid chains (C_16_–C_18_) connected through two weak ester bonds. Among the various applications, esterquats have been widely researched for their usage in fabric softener formulations.^13^

The softening action of surfactants relies heavily on their structure, with two alkyl chains and a positively charged hydrophilic group being crucial components. This makes esterquats a promising ingredient for fabric softener formulations.^12,14^ It represents a new generation of fabric softening agents, replacing traditional distearyl dimethylammonium compounds known for their poor biodegradability. Due to growing environmental concerns, most European fabric softener manufacturers have shifted away from dialkyl quats, favoring esterquats instead.^15^ This cationic esterquat surfactants exhibit strong biocidal activity due to the presence of two ammonium groups.^16^ They find significant application in the textile industry because of its bacteriostatic and fungicidal properties.^17^

Today, esterquats are recognized as a unique class of compounds with highly beneficial properties, showcasing their immense potential for various applications. Their popularity has significantly increased compared to a few decades ago, driven by the global shift toward a circular economy and efforts to protect the environment. Due to their potentially superior eco-toxicological profiles, particularly their susceptibility to biodegradation and lower toxicity to aquatic life, esterquats are gradually replacing some surfactants in both Europe and the United States. The growing interest in esterquats from major chemical companies like BASF, Henkel, Procter & Gamble, and Evonik highlights their significant usability and safety, which could potentially lead to widespread commercialization and large-scale production.^18^

**Figure 1:**
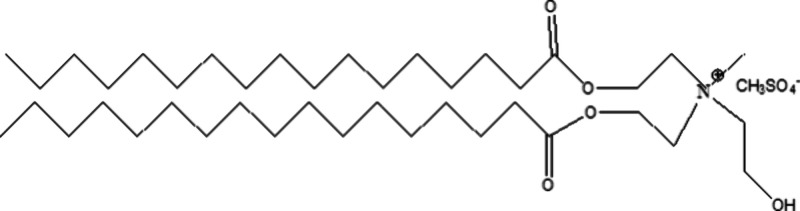
Chemical structure of esterquat.

In particular, cationic esterquat surfactants (Figure 1) have gained prominence for their eco-friendly, nontoxic, and biodegradable characteristics, making them highly suitable for applications in industries such as leather and textiles. Their unique structure, which includes ester linkages and quaternary ammonium groups, allows these surfactants to break down more easily in natural environments compared with traditional alternatives. Esterquats not only provide essential benefits such as softness, rewettability, and dispersing capabilities but also demonstrate excellent biodegradability, significantly reducing their ecological impact.^19^

The development of esterquats, biodegradable double-tailed quaternary ammonium surfactants featuring ester linkages, marks a significant advancement toward sustainability.^20,21^

The superior biodegradability of esterquats stems from their ester bonds, which are readily hydrolyzed by enzymes produced by microorganisms. This enzymatic process breaks down the ester bonds, releasing fatty acids and simpler compounds that microorganisms can further metabolize into carbon dioxide and water.^22^ Additionally, the presence of ester linkages between long hydrocarbon chains and triethanol hydroxyl groups enhances their biodegradability.^23^ Specifically, esterquat-based emulsions, which incorporate ester functionality in their spacers or headgroups along with quaternized entities, benefit from weaker linkages like ester bonds. This design leads to easily hydrolyzable moieties, further increasing their rate of biodegradation.^24,25^ Despite their numerous advantageous properties, there are limited studies on cationic fatliquoring agents developed using esterquats, and the use of esterquats in the leather industry remains quite limited.

In the present study, the aim was to develop an innovative and environmentally friendly cationic fatliquor emulsion for use in both the textile and leather industries, taking advantage of the unique properties of esterquat-based surfactants. Esterquats, known for their biodegradability, antibacterial activity, low toxicity, and excellent performance as softeners, were selected as the key components in the emulsion formulation. The cationic emulsion was designed to enhance the leather’s softness, handle, waterproofing, and fiber insulation and softening, while also contributing to an improved ecological footprint in textile and leather processing. This research aims to address the industry’s need for sustainable and high-performing softening agents, offering a cleaner production alternative that minimizes environmental impact while improving the physical properties of finished products. Additionally, the use of esterquat emulsions is expected to increase dyestuff exhaustion, further enhancing the leather’s aesthetic and functional qualities.

## Experimental Program

### Materials

Commercial domestic pickled sheepskins were used as the leather material for experiments. Oleic acid, Tween 60, Tween 80, Span 60, polydimethylsiloxane, and pH adjusters used in the production of cationic lubricating emulsion were obtained from Sigma-Aldrich. Esterquat was purchased from Ataman Chemicals. Cotton and polyester fabrics were obtained from SKU Auezov University.

### Methods

#### Emulsification Studies

In the production of an oil-in-water (O/W) emulsion, oleic acid, Span 60, esterquat, and dimethylsiloxane were combined in a beaker to form the oil phase. Separately, Tween 60 was dissolved in water to create the aqueous phase, forming a pre-emulsion in a reaction flask. The oil phase was subsequently added to the pre-emulsion, and the mixture was subjected to high-speed stirring for 30 min, resulting in the formation of an O/W emulsion. To enhance the homogenization of emulsion droplets and reduce particle size, the mixture was ultrasonicated in an ice bath for 15 min. The pH of the emulsion was adjusted to 4.5 during the homogenization process. The hydrophilic–lipophilic balance (HLB) of the emulsion was optimized to 7.3 using a combination of Span 60 (HLB 4.7) and Tween 60 (HLB 14.9). After preparation, the emulsions were cooled to room temperature, and the resulting emulsion exhibited good dispersibility in water without the signs of phase separation or instability.

#### Particle Size and Zeta Potential Analysis

The particle size and zeta potential of the prepared fatliquors were analyzed using a Malvern Zeta Sizer Nano ZS, which has a measurement range of 0.1–10,000 nm. For the analysis, the emulsion samples including oleic acid, Span 60, Tween 60, and polydimethylsiloxane were prepared at a concentration of 0.1 mg/mL by diluting the original emulsions with deionized water. This method allowed for the accurate characterization of the emulsion’s particle size distribution and surface charge, providing insights into the stability and dispersion behavior of the fatliquor in aqueous systems.

#### Leather Production

In leather production, wet processing steps are carried out in conventional drum systems, which serve as both reaction vessels and mechanical processing units. These drums are designed to facilitate the penetration of chemicals into the leather matrix and promote the chemical reactions necessary for leather transformation.

The process involves the use of a high volume of water, combined with the rotational motion of the drum, to generate a mechanical action. This mechanical action ensures a uniform distribution of chemicals across the leather surface and enhances their penetration into the deeper layers of the skin. Under atmospheric pressure, the added chemicals interact with the leather matrix, promoting reactions such as collagen stabilization, retanning, dyeing, and fatliquoring. The controlled conditions within the drum, including temperature, pH, and processing time, are critical to achieving the desired physical and chemical properties in the final leather product.

The production recipe for wet-blue (chrome-tanned) leathers is outlined in Table 1, covering the processes of neutralization, dyeing, fatliquoring, and retanning. The leather production recipe mainly begins with a neutralization process, where water is added to the drum at 100% of the leather’s weight. Following this, 1.5% sodium formate is introduced, and after initial mixing, 0.8% sodium bicarbonate is gradually added to adjust the pH to 5.5. The drum is run for two 30 min cycles to ensure uniform neutralization across the leather. After this step, the drum is drained and the leather is rinsed twice with fresh water to remove residual chemicals. The retanning, dyeing, and fluorescence processes follow. Initially, 80% water is added to the drum and heated to 40 °C. Once the temperature stabilizes, 3% acrylic syntan is introduced, and the drum is run for 30 min. Subsequently, 3% melamine resin and 3% phenolic syntan are added in succession, each step accompanied by an additional 30 min rotation to ensure thorough penetration of the chemicals. For dyeing, 3% dyeing auxiliary and 4% acid dye are mixed with water at 40 °C. This process continues for 30 min to achieve uniform dye uptake. After dyeing, 50% additional water is introduced, and the temperature is raised to 50 °C. The fatliquoring process then begins with the addition of a mixture of 3% lecithin-based fatliquor, 3% sulfite fish fatliquor, and 2% synthetic fatliquor, involving a 60 min drum rotation. Then, the pH is adjusted to 4.0 using formic acid for fixation of the chemicals. At this stage, cationic fatliquoring emulsions are applied at variable concentrations of 0.5, 1, 2, 3, and 4%. The drum is run for 60 min to allow the emulsions to penetrate the leather fibers thoroughly, enhancing softness and other physical properties, and the leather is rinsed with water. Finally, the process concludes with overnight horsing-up, during which the excess water drains from the leather and the material stabilizes.

**Table 1:** Leather Production Recipe

process	%	chemicals	temperature (°C)	time (min.)	remarks
washing	200	water	30		
	2	acetic acid		40	drain
neutralization	100	water	30		
	1.5	sodium formate		30	
	0.8	sodium bicarbonate		2 × 30.	pH 5.5, drain
washing 2×	200	water	30		drain
retanning, dyeing, fatliquoring	80	water	40		
	3	acrylic syntan		30	
	3	melamine resin		30	
	3	phenolic syntan		30	
	3	dyeing auxiliary		30	
	4	acid dye		30	
	+50	water	50		
	3	lecithine-based fatliquor			
	3	sulfite fish fatliquor			
	2	synthetic fatliquor		60	
	2	formic acid		40	pH 4
	*	cationic fatliquoring emulsions		60	
drain–washing–horsing up overnight					

The key focus in this recipe is the use of a cationic fatliquoring emulsion at varying concentrations, specifically 0.5, 1, 2, 3, and 4%. This stepwise variation aims to evaluate the impact of different emulsion concentrations on the physical properties of the finished leather, such as softness, water resistance, and tensile strength, while also assessing the overall performance of the fatliquor in improving leather quality during the post-tanning processes. By comparing these treated samples against the untreated control, we were able to demonstrate the specific contributions of the emulsion to leather performance.

#### Textile Application Processes

Following the completion of pretreatment, dyeing, and washing processes for both cotton and polyester fabrics, cationic emulsions were applied using the exhaust method. This method involves immersing fabrics in a treatment bath containing the cationic emulsion under controlled conditions of the temperature, pH, and agitation. This method was chosen due to its effectiveness in ensuring uniform application, deep penetration, and efficient fixation of the emulsion on the fabric fibers. The treatment was conducted at a temperature of 40–45 °C for 30 min, allowing the emulsion to thoroughly penetrate and interact with the fiber surfaces.

After the emulsion application, the fabrics were dried at 100 °C for 5 min, followed by curing in a stenter at 120 °C for 30 s. The drying step removed excess moisture, while the curing process ensured strong fixation of the emulsion components onto the fibers. The curing at elevated temperatures enhanced the durability of the treatment, ensuring long-lasting softness and performance.

#### Softening Power (Subjective Evaluation)

The softening power of the treated fabrics was evaluated subjectively through panel feel test conducted by trained panelists as follows.^26^

##### Fabric Sample Preparation

Fabric samples are prepared, including both untreated and treated specimens, to evaluate the effects of the softening agent.

##### Sensory Evaluation

A trained group of panelists assesses the softness of the fabric through tactile interactions. The panelists perform various manipulative techniques, such as squeezing, twisting, and rubbing, to test the softness of the fabric samples.

## Results

A scoring system is implemented based on the softness level observed, typically ranging from 1 to 5 where 5 represents the highest level of softness, to quantify the effectiveness of the softening treatment. This score reflects the panelists’ subjective evaluations, providing a comprehensive assessment of the fabric’s tactile qualities.

### Physical Properties of Treated Leathers

**Figure 2:**
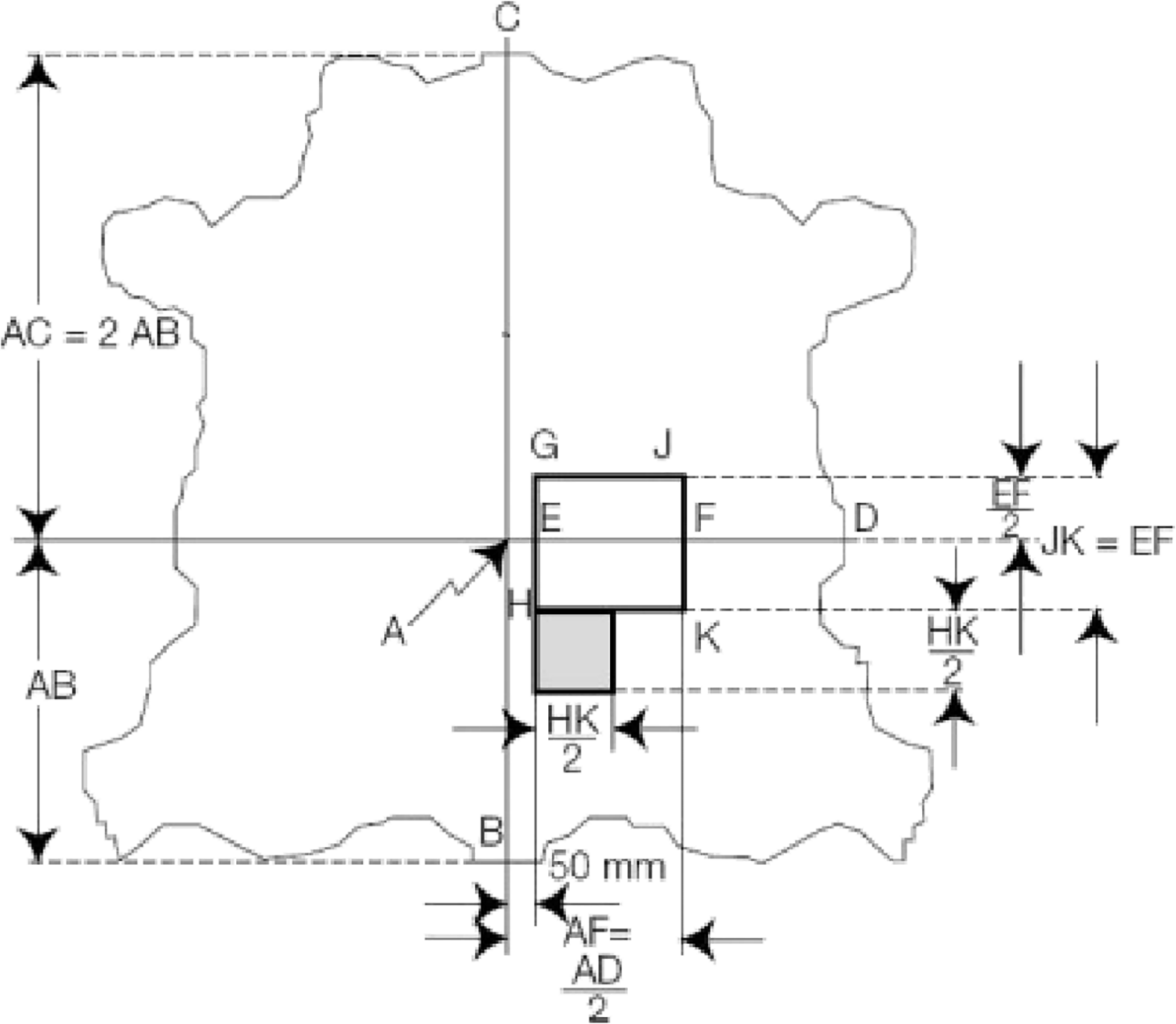
Sampling the HGJK square for physical tests.

Prior to analysis, all skin samples were conditioned (EN ISO 2419) and then sampled as shown in Figure 2 (EN ISO 2418).

The determination of the tensile strength and elongation percentages in leather samples was performed in accordance with the TS EN ISO 3376 standard testing methodology. Single-edge tear load variations in the finished leathers were quantified using TS EN ISO 3377-1 Tear Load Determination 1: Single-Edge Tear standard, utilizing the Shimadzu AG-IS testing machine for precise measurement. Similarly, the evaluation of double-edge tear load variations in the leather samples was carried out following the guidelines set forth in TS EN ISO 3377-2 Tear Load Determination 2: Double-Edge Tear standard. To assess the filling effect (%) of the prepared emulsions on the leather, the wet thickness of the treated leather samples was compared against that of the control group samples. The percentage increase in the leather thickness was subsequently calculated. After the application of the recipe detailed in Table 1, the leather samples were removed from the conditioning chamber, excess moisture was allowed to drain, and the determination of leather thickness was conducted in accordance with TS 4117 EN ISO 2589. The analyses were executed in triplicate, and the resultant thickness increases were expressed as percentages to provide a comprehensive assessment of the emulsion’s efficacy.

### Antibacterial Activity Tests

In this study, the antibacterial properties of fabric treated with the cationic fatliquoring emulsion were evaluated in accordance with the AATCC 100 standard, while the antibacterial properties of leather were assessed by using the ASTM E 2149-01 standard. These standards were employed to ensure a reliable and standardized method for determining the antimicrobial activity of treated materials under controlled conditions. *Staphylococcus aureus* (ATCC 29213) as gram-positive bacterial strain and *Escherichia coli* (ATCC 25922) as gram-negative strain were used in the evaluation of antibacterial efficacy.

The antibacterial activity of the treated fabric was evaluated by following the AATCC 100 standard. Fabric samples with diameters of approximately 5 cm were cut and sterilized prior to testing. To calculate recovery, an untreated fabric sample or a sample with a contact time of 0 min was used as the control. *S. aureus* (ATCC 29213) and *E. coli* (ATCC 25922) were incubated on agar media at 37 ± 2 °C for 24 h.

From the 24 h culture, a bacterial suspension was prepared in physiological saline to reach a concentration of 1–2 × 10^5^ cfu, ensuring a homogeneous mixture. A 1 mL aliquot of the bacterial suspension was then applied to the surface of the fabric samples, which were subsequently incubated at 37 °C for 24 h. Immediately after inoculation, one sample was transferred into a bottle containing 100 mL of a neutralizing agent (Lecithin and Tween 80), while other samples were transferred after the incubation period. The bottles were shaken vigorously for 1 min to ensure proper mixing, and serial dilutions were prepared.

From each dilution, 1 mL was plated on Petri dishes, followed by the addition of approximately 15 mL of Tryptic Soy Agar (TSA). Once the agar solidified, the plates were incubated at 37 °C for 24 h. After incubation, colonies were counted, and the reduction in bacterial count was calculated using the following formula:
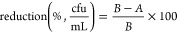
where *B* represents the number of bacteria recovered from the untreated control sample immediately after inoculation and *A* represents the number of bacteria recovered from the treated fabric sample after incubation.

For leather antibacterial assessments; initially, the bacterial inoculum was prepared by diluting to the 0.5 McFarland turbidity standard, equating to a concentration of 1.5–3.0 × 10^8^ cfu/mL, using sterilized Ringer solution. To verify the bacterial concentration, spectrophotometric measurements were taken at 625 nm. The bacterial solution was further diluted in a sterile buffer (0.3 mM KH_2_PO_4_, pH 7.2 ± 0.1) to a final concentration of 1.5–3.0 × 10^5^ cfu/mL, forming the working bacterial solution for the assay.

For the antimicrobial activity test, 2 cm × 2 cm fatliquored leather samples were placed in a sterile 250 mL flask containing 50 mL of the prepared bacterial solution. The flask was then incubated at 37 °C under orbital stirring. After 1 h of incubation, 1 mL of the solution was aseptically collected, and the bacterial concentration was determined via standard plate counting on nutrient agar. Colony forming units per milliliter (cfu/mL) were calculated, and bacterial reduction was expressed as a percentage (%).

The percentage of colony number reduction was determined according to the following equation.
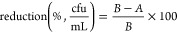


*B* – *A* = death rate constant

*A* = cfu per milliliter for the flask containing the treated substrate after the specified contact time

*B* = “0” contact time cubic units (cfu) per milliliter for the flask used to determine *A* before the addition of the treated substrate.

### Determination of Water Repellence Properties

The leather samples required for the test were cut into dimensions of 60 × 75 mm in accordance with TS EN ISO 5403-1. The rectangular leather specimens were horizontally fixed between two coaxial metal cylinders, with half of the outer surface of each leather piece submerged in deionized water. The setup was subjected to an oscillatory motion.

During the test, the amount of water absorbed by the leather samples was determined as a percentage. To calculate this, the leather samples were weighed prior to testing (W1) and reweighed after 1 h of testing (W2). The percentage of water absorption was calculated using the following formula:
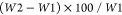
where *W*2 represents the weight of the sample after testing and *W*1 represents the weight of the sample before testing.

### UV-Spectrophotometer Analysis of Dyestuff Concentration

**Figure 3:**
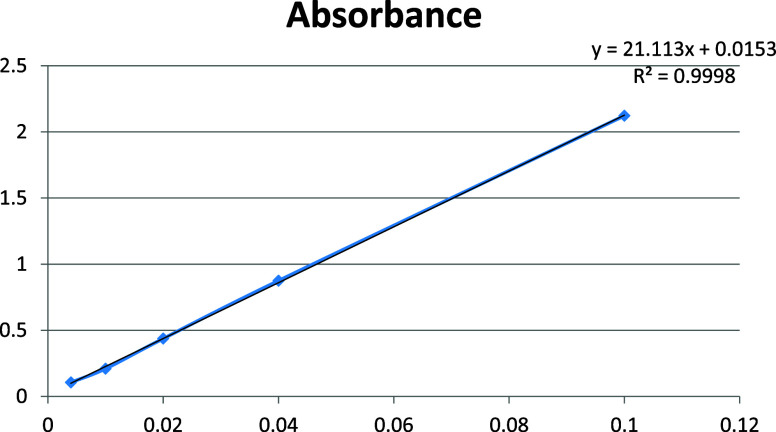
Concentration–absorbance graph of different ratios of dyestuff solution.

The effect of cationic emulsion used in leather production recipes at 0.5, 1, 2, 3, and 4% on dye consumption was investigated in a Shimadzu UV-1800 UV–visible Spectrophotometer. The absorbance differences were compared to the absorbance value of the dyestuff itself before dyeing, and the necessary dilutions were made in order to operate in a healthy reading range in the device. According to these measurements, a reference calibration curve was created, and absorbance and concentration values were determined (Figure 3).

Dyestuff exhaustion (E%) was calculated according to the following formula:
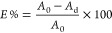
where *A*_0_ is the absorbance values of the dye solution in the liquor before dyeing and *A*_d_ is the absorbance values of the dye solution in liquor after dyeing. Measurements were taken at the wavelength at which the dye solution showed maximum absorbance (λ_max_).

## Results and Discussion

### Particle Size and Zeta Potential of the Emulsion

The average particle size and zeta potential are fundamental parameters in assessing the colloidal stability of emulsions, as they directly influence emulsion behavior, including coalescence and aggregation tendencies.^27^ In leather production, the efficiency of fatliquoring agents is strongly dictated by their particle size, which determines the degree of lubrication imparted to the leather matrix. Smaller particle sizes in fatliquor emulsions significantly increase the specific surface area per unit volume, thereby enhancing the interface available for interactions with leather fibers. This enlarged surface area not only promotes more efficient chemical binding but also facilitates deeper and more uniform penetration of the emulsion into the fibrous leather network, ultimately improving the overall lubrication and functional performance of the leather.^28^ The average particle size and zeta potential values of the formulated fatliquor emulsion are presented in Table 2.

**Table 2:** Particle Size and Zeta Potential of the Emulsion

	average size diameter (μm)	zeta potential (mV)
emulsion	5.52	+58.4

The results from the zeta potential analysis, showing a ζ-potential of +58.4 mV at pH 4.5, indicate that the emulsion exhibits strong colloidal stability. Typically, zeta potential values above ±30 mV suggest that an emulsion has sufficient electrostatic repulsion to resist aggregation, ensuring a more stable dispersion over time.^29^ In this case, the high positive zeta potential suggests that the emulsion droplets are well dispersed and unlikely to coalesce, thus maintaining stability under acidic conditions (pH 4.5), which is relevant in leather processing environments. This stability is particularly beneficial in textile softening applications, where uniform dispersion of the softening agent across the fabric surface is key to achieving consistent softness.^30^

The average particle size of 5.52 μm places the emulsion in the microscale range. While smaller particle sizes (in the submicron range) would provide a larger surface area and more efficient interaction with leather fibers, a particle size of 5.52 μm is still within an acceptable range for effective fatliquoring.^5,11^ This size allows for good penetration into the leather matrix and adequate surface interaction to facilitate lubrication.^31^ Together, the zeta potential and particle size results suggest that the emulsion is well suited for leather fatliquoring, offering both stability and effective lubrication, although there may be room for optimization to further improve the performance. Moreover, during the study, no stability problems such as phase separation and creaming were encountered in the emulsion produced as an oil-in-water emulsion (O/W). The color of the prepared emulsion was determined as yellowish white, and no color change was observed during the experiments.

### Softening Power Test Results

Softening finishes represent a critical category of chemical treatments in the textile industry. The application of chemical softeners allows textile products to attain desirable characteristics, such as an acceptable soft hand, enhanced smoothness, increased flexibility, and improved drapability and pliability.^32^ Numerous types of softeners with varying chemical structures exist, among which cationic softeners are recognized as one of the most significant classes due to their effective performance and compatibility with various fabric types.^33^

The prepared cationic emulsion was applied to both cotton and polyester substrates at varying concentrations of 0.5% (owf), 1% (owf), 2% (owf), and 3% (owf) using the exhaust method. This range of dosages was selected to evaluate the softening effect at different levels of emulsion concentration. The test aimed to assess the impact of each dosage on the softening efficiency of the fabric by analyzing factors such as hand feel, drapability, and surface smoothness. Comparative results between the cotton and polyester substrates provide insight into the emulsion’s effectiveness across different fiber types.

The softness of the treated fabrics was evaluated by using a panel feel test, where a group of experienced assessors qualitatively assessed the softness of each sample. The fabrics were rated on a scale from 1 to 5, with a rating of 5 indicating the highest level of softness and 1 representing the lowest or poorest, softness. The results of this assessment are presented in Table 3, providing a comparative analysis of the softness achieved at different emulsion dosages and for both cotton and polyester substrates.

**Table 3:** Softness Characteristics of Cotton and Polyester Fabrics Treated with Cationic Emulsions

	**softness rating**				
	**dosage (%)**				
**fabrics**	0.5	1	2	3	commercial softener (%3)
cotton	3	3	3.5	4.5	5
polyester	2	2	3	3	4

The results presented in Table 3 illustrate the effectiveness of cationic emulsions in enhancing the softness of cotton and polyester fabrics. As the concentration of the softener increases, a notable improvement in the softness is observed for both fabric types. This softening mechanism of cationic emulsions relies on the action of cationic surfactants, particularly esterquats, which play a dual role in enhancing the properties of fabrics. These surfactants adsorb onto the surface of textile fibers due to their positive charge, which facilitates strong electrostatic interactions with the negatively charged fiber surface. Upon adsorption, their hydrophobic groups align outward away from the fabric surface, creating a lubricating layer. This arrangement significantly reduces the friction between the individual fibers, leading to a smoother texture. Additionally, the hydrophobic orientation not only prevents static cling by dissipating electrical charges but also contributes to the perception of softness and a fluffy, voluminous feel, improving both the tactile and aesthetic qualities of the fabric.^19^ For cotton, even at a lower concentration of 0.5%, the softness rating is moderate at 3, but it steadily increases as the dosage rises, reaching a near-maximum softness of 4.5 at a 3% concentration. This demonstrates that the softening power of the emulsion is dosage-dependent, with higher concentrations leading to significant enhancements in the hand feel and surface smoothness of the cotton fabric. The performance of the emulsion on cotton at 3% is only slightly lower than that of the commercial softener, which achieves a perfect softness rating of 5.

Polyester fabric, on the other hand, shows less sensitivity to the lower concentrations of the softener, with ratings of 2 for both the 0.5 and 1% dosages. However, as the concentration increases to 2 and 3%, the softness ratings improve to 3, suggesting that polyester may require higher concentrations of the cationic emulsion to achieve a noticeable improvement in softness. The commercial softener shows a superior performance on polyester with a softness rating of 4, which is higher than the 3% cationic emulsion’s effect but still demonstrates that polyester benefits less from softeners than cotton does.

### Physical Properties of Treated Leathers

Fatliquors are essential agents in leather processing that play a critical role in improving tear strength and enhancing other key physical properties such as flexibility, elongation, and tensile strength.^11^ Their primary mechanism of action involves penetrating the fibrous structure of leather and depositing a thin lubricating layer around the fibers and fibrils. This lubrication prevents the fibers from adhering to one another during the drying phase, thereby maintaining the structural integrity of the leather. Once the leather achieves its equilibrium moisture content, the lubricated fibers are able to slide over one another smoothly, allowing for better stress distribution under mechanical forces.^4^ The resulting sliding action contributes to the overall flexibility and softness of the leather, facilitating the alignment of fibers in accordance with the applied strain in any direction. The quantity and type of fatliquor emulsion used significantly influence various physical properties of leather, including fullness, tensile strength, tear strength, and elongation, as well as permeability to water, air, and water vapor. Additionally, these factors impact water absorption.^34^ The results of the analyses, including filling efficiency, tensile strength, elongation at break, single-edge tear load, and double-edge tear load for the fatliquored leathers, are presented in Table 4.

**Table 4:** Effect of Fatliquoring Emulsion Concentration on Leather’s Physical Properties

	**leather’s physical properties**									
**concentration (%)**	**tensile strength**(N/mm^2^)		**elongation (%)**		**single-edge tear load (N)**		**double-edge tear load (N)**		**filling efficiency (%)**	
	mean + SD^a^	**SE^b^**	mean + SD	**SE**	mean + SD	**SE**	mean + SD	**SE**	mean + SD	**SE**
control	12.87 ± 0.52	0.29	41 ± 1.60	0.92	7.89 ± 0.45	0.26	12.29 ± 0.68	0.39		
0.5%	13.52 ± 0.68	0.39	37 ± 1.85	1.07	9.80 ± 0.49	0.28	15.70 + 0.79	0.45	6.2 ± 0.4	0.23
1%	13.90 ± 0.77	0.44	42.23 ± 2.32	1.34	10.25 ± 0.56	0.33	16.29 ± 0.90	0.52	6.5 ± 0.44	0.25
2%	13.53 ± 0.81	0.47	44.18 ± 2.65	1.53	11.97 ± 0.72	0.42	17.87 ± 1.07	0.62	7.25 ± 0.48	0.27
3%	13.68 ± 0.89	0.51	48.6 ± 3.16	1.82	12.05 ± 0.78	0.45	17.75 ± 1.15	0.67	7.05 ± 0.52	0.30
4%	13.59 ± 0.95	0.55	46.16 ± 3.23	7.87	12.52 ± 0.87	0.50	17.88 ± 1.25	0.72	6.52 ± 0.55	0.32

a Standard deviation.

b Standard error.

Table 4 provides a comparative analysis of the physical properties of leathers treated with different concentrations of a fatliquor emulsion (0.5, 1, 2, 3, and 4%) against a control group, which did not undergo any emulsion treatment. The comparison of the treated samples to the control group highlights the impact of the emulsion on leather performance, allowing for a clearer understanding of how fatliquoring affects the leather’s physical characteristics.

The results presented in Table 4 demonstrate a clear enhancement in the physical and mechanical properties of leathers treated with the fatliquoring emulsion compared to those of the control group. These findings align with previous studies on the impact of fatliquoring on leather strength.^35−37^ Specifically, the data shows an increase in tensile strength and tear resistance as the concentration of the fatliquor emulsion increases, which is consistent with the hypothesis that fatliquoring not only lubricates but also reinforces the leather structure.^38^

In the tensile strength analysis, the untreated control group exhibits a baseline strength of 12.87 ± 0.52 N/mm^2^ N/mm^2^, which is notably lower than all fatliquored samples. The highest tensile strength (13.90 ± 0.77 N/mm^2^) is observed at a 1% emulsion concentration. After this peak, the tensile strength values fluctuated slightly, showing a minor decline at higher concentrations with 13.59 ± 0.95 N/mm^2^ observed at 4%. This initial increase can be attributed to the effective exhaustion of anionic fatliquoring blend and lubrication provided by the fatliquor, which enhances the alignment of collagen fibers under applied stress. The subsequent decrease at higher concentrations likely results from overlubrication, which may reduce fiber-to-fiber interaction, diminishing the mechanical strength.

Tensile strength varies according to the type of leather, sex of the animal, type of tanning, and other chemicals used in production. Tanning and retanning agents encourage the formation of new additional bonds between the fibers in the fiber structure of the leather and increase the adhesive strength of the fibers, which increases the tensile strength of the fibers.^39^ The moisture content of the leather also has a direct effect on the tensile strength. When the leather is wet, the cohesion forces in the bonds between the elements forming the fiber weave are not very strong. For this reason, collagen fibers and fiber bundles slide over each other. While this situation decreases the tensile strength, it increases the tear strength.^37,38^ One of the most important processes affecting the strength properties of leather products is the retanning–fatliquoring process. Especially in the fatliquoring process, the low water content of the leather after tanning can be compensated by dosing high fat content, which facilitates the sliding of the fibers on each other and improves the strength values.^40^

Findings from Table 4 suggest that the optimal concentration for improving tensile strength is around 1% for cationic application, balancing lubrication, and fiber cohesion. This trend indicates that the introduction of fatliquors at a certain level into the leather’s matrix enhances the internal lubrication of collagen fibers, enabling them to slide more effectively over each other under stress.^35^ Similar findings were observed by ref (41), who noted that fatliquoring may reduce the strength of individual fibers but increases the overall bulk leather strength by improving the lubrication between fibers, allowing a more uniform distribution of tensile forces. This uniform distribution lowers internal stress within the fiber network, ultimately making the leather stronger under applied forces.

Elongation behavior exhibited a unique trend, initially decreasing at 0.5% concentration (37 ± 1.85%) compared to the control (41 ± 1.60%) and then increasing steadily, peaking at 48.6 ± 3.16% at 3% concentration. A slight decrease to 46.16 ± 3.23% was observed at 4%. The initial decrease at lower concentrations may be due to insufficient lubrication, which restricts fiber movement. As the alcohol concentration increases, the improved lubrication facilitates greater fiber mobility and realignment under stress, enhancing elongation.

The most striking enhancement was determined in the tear strength values. The single-edge tear load exhibited a consistent increase across all tested fatliquoring emulsion concentrations, starting from 7.89 ± 0.45 N in the control group and reaching a maximum value of 12.52 ± 0.87 N at 4% concentration. This marks an improvement of over 58% compared to that of the untreated leather. The double-edge tear load followed a similar trend, improving consistently from 12.29 ± 0.68 N in the control group to 17.88 ± 1.25 N at 4% concentration, representing an increase of approximately 45%. This significant enhancement can be attributed to the lubricating effect of the fatliquoring emulsion, which reduces internal friction within the leather matrix. By creating a lubricated interface between collagen fibers and fiber bundles, the fatliquor facilitates smoother sliding and redistribution of stress under tearing forces. This reduction in localized stress concentrations prevents fiber breakage and increases the leather’s resistance to tearing.^36^

Tear strength is a critical mechanical property that influences the usability of leather. In some instances, insufficient tear strength can pose significant challenges. For example, during the shoe-making process, leather is stretched into a shape, which can result in small cracks, tears, or holes. These imperfections can negatively impact the appearance, durability, and lifespan of the shoes.

Fatliquoring is recognized as one of the most crucial processes for enhancing the tear strength in leather. During fatliquoring, the oil emulsions penetrate the fibrous structure of the leather, increasing its moisture content, preventing adhesion between the fibers and facilitating their sliding over each other. These mechanisms collectively contribute to improved tear resistance. The effectiveness of the fatliquoring process increases as the particle size of the oil emulsions decreases. Natural oils such as lanolin, lecithin, and fish oil help regulate the moisture level of the leather, while the use of long-chain, high-emulsifier content fatliquoring agents further enhances fiber isolation, thereby improving tear strength.^37,38^

The observed improvement in tear strength in our study can be directly linked to the mechanisms described in the provided explanation of fatliquoring. Our emulsion, composed of oleic acid, esterquat, polydimethylsiloxane, and emulsifiers, exhibits characteristics that align well with these principles. Oleic acid, a long-chain fatty acid, plays a pivotal role by deeply penetrating the leather matrix and increasing its moisture content, reducing internal friction and facilitating the sliding of collagen fibers under stress. This mechanism ensures a more uniform distribution of tearing forces, enhancing both single-edge and double-edge tear resistance.^42^

Esterquat, as a cationic surfactant, strengthens the interaction between the lipid and the negatively charged leather matrix. This strong electrostatic attraction ensures uniform distribution, exhausting anionic chemicals, and deep penetration of the emulsion. This reduces fiber entanglement and enhances the structural cohesion, leading to greater resistance against tearing forces. Polydimethylsiloxane contributes to maintaining the leather’s optimal moisture balance, reinforcing lubrication, and preventing excessive fiber saturation. Its ability to improve fiber pliability and resist tearing further supports the mechanical integrity of the leather.^43^

Finally, emulsifiers such as Span 60 and Tween 60 stabilize the emulsion, enabling smaller particle sizes that penetrate deeper into the matrix and ensure consistent lubrication and stress redistribution. This combination of penetration, lubrication, and cohesion is critical to the enhanced tear resistance observed in the study.

As reported by ref (38), this increase in tear resistance can be attributed to the improved lubrication between the fibers, which allows for a more even distribution of stress during tearing, thus reducing the possibility of fiber rupture.

Filling efficiency results showed a consistent increase from 6.2 ± 0.4% at the 0.5% concentration to 6.5 ± 0.44% at 1%, and peaking at 7.25 ± 0.48% at 2%. Beyond this point, a slight decrease was observed with 7.05 ± 0.52% at 3% and 6.52 ± 0.55% at 4%. These results indicate that the highest fullness was achieved at 2%, suggesting optimal penetration and distribution of the fatliquoring emulsion within the leather matrix at this concentration. Such improvements in fullness are critical in ensuring a more luxurious feel and better mechanical properties for the final leather product.

### Antibacterial Test Results

In today’s world, where environmental and health concerns are prioritized, biodegradable products such as esterquats are emerging as promising softening agents. Their antimicrobial properties make them an important alternative for the textile and leather industries, contributing to sustainable practices while enhancing product performance.^44^ Esterquats, a class of cationic surfactants derived from fatty acids and quaternary ammonium compounds, are known for their antibacterial properties, which make them valuable in various applications, particularly in textile applications.^45^ The antibacterial effects of the materials treated with cationic emulsion consisting of esterquat, oleic acid, and polydimethylsiloxane with different percentages are shown in Tables 5 and 6.

**Table 5:** Reduction of Microorganism Growth on Fabrics with Cationic Emulsion

	E. coli reduction rate (%)-AATCC 100 Standard					S. aureus reduction rate (%)-AATCC 100 Standard				
	**dosage (%)**									
**materials**	0.5	1	2	3	commercial softener (%3)	0.5	1	2	3	commercial softener (%3)
cotton	11.25	18.74	22.48	26.57	0	13.45	18.52	31.25	32.53	0
polyester	8.45	12.23	12.48	17.86	0	12.54	17.59	23.45	28.97	0

**Table 6:** Reduction of Microorganism Growth on Leather with Cationic Emulsion

	E. coli reduction rate (%)-ASTM E 2149-01 Standard					S. aureus reduction rate (%)-ASTM E 2149-01 Standard				
	**dosage (%)**									
**materials**	0.5	1	2	3	Control	0.5	1	2	3	control
leather	25.41	28.98	41.52	57.83	0	42.54	44.85	68.63	79.46	0

Table 5 provides the results of the antibacterial test on cotton and polyester fabrics treated with cationic emulsions at varying dosages (0.5, 1, 2, and 3%) and compares their effectiveness in reducing the growth of *E. coli* and *S. aureus* to a commercial softener (3%). The reduction rates of microorganism growth are reported according to the AATCC 100 standard. The antibacterial activity of the emulsion is primarily derived from the presence of esterquats, which play a crucial role in microbial inactivation. The antimicrobial activity of esterquats is primarily related to their cationic surfactant properties. The mechanism involves the adsorption of esterquat molecules onto the surface of the microbes followed by their penetration into the microbial cell membrane. This interaction disrupts the membrane structure, compromising its integrity and leading to cell death. The bactericidal efficiency of esterquats is also influenced by several factors, including the length of their hydrophobic chains, surface activity, the type of alkylation agent used, dosage levels, and the structural characteristics of the bacterial cell membrane.^46^ The main mode of antimicrobial action of esterquat is due to electrostatic interaction with cell membranes, predominantly at the level of the cytoplasmic membrane causing disruption and leakage of the cellular content.^44^ On the other hand, oleic acid contributes to the antibacterial activity by integrating into the lipid bilayer of bacterial membranes, causing structural disruption. This amphiphilic molecule can destabilize the membrane integrity, further enhancing the bactericidal effect of esterquat.^47,48^

The commercial softener, tested at 3%, exhibited no significant antibacterial activity for either *E. coli* or *S. aureus*. This is likely due to its composition being optimized for fabric softening rather than antimicrobial properties. In contrast, the cationic fatliquor emulsion demonstrates clear antibacterial activity due to its functional components, particularly esterquat and oleic acid, which directly target microbial growth.

For cotton fabrics, the cationic emulsion demonstrated moderate antibacterial activity, especially at higher concentrations. At a 0.5% dosage, the reduction of *E. coli* is relatively low at 11.25% but increases to a significant 26.57% at 3%. Similarly, the reduction of *S. aureus* shows a steady increase with higher dosages, starting at 13.45% for 0.5% and reaching 32.53% at 3%. This difference in antibacterial efficacy can be attributed to the structural characteristics of Gram-negative bacteria, whose outer membrane is predominantly composed of lipopolysaccharides and proteins that restrict the penetration of biocides and amphiphilic compounds, rendering them more resistant than Gram-positive bacteria.^49^

For polyester fabrics, the effectiveness of the cationic emulsion is slightly lower compared to that of cotton, particularly for *E. coli*. At 0.5%, the reduction rate is only 8.45%, increasing to 17.86% at 3%. The results for *S. aureus* are more promising, with a reduction of 12.54% at 0.5% and an improvement to 28.97% at 3%. Polyester’s inherent properties might explain the lower overall antibacterial activity, as synthetic fibers are less absorbent, potentially limiting the penetration of the emulsion into the fabric structure compared to natural cotton fibers. Polyester, being a synthetic material, has a smoother and less porous structure, which limits the absorption and penetration of the emulsion into the fabric. In contrast, cotton’s natural, more absorbent and hydrophilic structure allows for better emulsion uptake, facilitating a stronger interaction with the fiber. Additionally, cotton fibers have functional groups, like hydroxyl groups, that can bond more effectively with the cationic components of the emulsion, enhancing its antibacterial properties.^50^ Polyester lacks these reactive sites, making the adhesion of the emulsion less efficient. Furthermore, the electrostatic attraction between the cationic emulsion and the fiber surface is likely stronger in cotton than in polyester, leading to better coverage and distribution of the emulsion in cotton fabrics.^51^ These factors collectively result in lower antibacterial effectiveness of the emulsion on polyester fabrics. Upon examination of the antibacterial activity results of the fabric samples presented in Table 5, it is evident that the fabric sample treated with cationic emulsion exhibited a measurable level of antibacterial activity against both tested bacterial strains; however, this activity was not particularly high. The enhancement of this antibacterial activity could potentially be achieved by increasing the concentration of the applied emulsion. However, it is important to note that higher concentrations of the softener may lead to yellowing of the fabric due to the inherent yellowish hue of the emulsion used. Therefore, for textile surfaces where color is not a critical factor, or for those with darker backgrounds, it would be feasible to increase the concentration of the softener to achieve surfaces with improved antibacterial properties.

Table 6 presents the antibacterial activity of leathers treated with cationic emulsions at varying concentrations (0.5, 1, 2, and 3%), compared to an untreated control group. The effectiveness of the emulsion is measured by the reduction in *E. coli* and *S. aureus* growth, following the ASTM E 2149-01 standard. A reduction in microorganisms above 75% is considered to be effective in demonstrating antibacterial activity.

For leathers treated with the cationic emulsion, the reduction of *E. coli* shows a clear dose-dependent trend, starting at 25.41% for 0.5% emulsion and reaching 57.83% at 3%, far exceeding the control group, which shows no antibacterial effect. The increase in the reduction rates with higher concentrations demonstrates that the emulsion effectively penetrates the leather matrix and inhibits bacterial growth. However, the results suggest that while the reduction rate is significant, it falls short of the 75% threshold necessary to confirm potent antibacterial action against *E. coli* at all concentrations. In contrast, the emulsion exhibits much stronger antibacterial activity against *S. aureus*. At a 0.5% dosage, the reduction rate starts at 42.54%, rising to 79.46% at 3%. This exceeds the 75% threshold, confirming the emulsion’s effectiveness in providing antibacterial protection against *S. aureus* at higher concentrations.

It is worth noting that the difference in antibacterial effectiveness between *E. coli* and *S. aureus* aligns with prior research, indicating that Gram-negative bacteria like *E. coli* tend to be less sensitive to antimicrobial agents than Gram-positive bacteria due to the structural differences in their cell membranes. As Gram-negative bacteria have an additional outer membrane, they are more resistant to this disruption compared to Gram-positive bacteria.^52^

### Water Repellence Test Results

Under standard conditions, leather possesses a natural fiber weave structure, enabling limited moisture absorption. Consequently, leather products, especially those used in winter, can become damp in humid environments. To maintain strength and shape, it is essential to restrict leather’s water absorption capacity, making its water resistance properties critical for hygiene and physiological protection during use. Various methods are employed during production to enhance the hydrophobic characteristics of leather to meet consumer demands. Achieving optimal specifications for high water resistance and durability requires a balanced combination of hydrophobic and hydrophilic components in the treatment applications. Lubricants play a vital role in minimizing water absorption and in promoting hydrophobic properties. Given that hydrophobic leather production occurs in aqueous environments, the chemicals used must be water-soluble and contain hydrophilic groups for compatibility, alongside hydrophobic groups to effectively reduce water absorption.^53^ The lubricating emulsions bond with the leather substrate through reactive groups, allowing for controlled water absorption. During this process, oil-in-water emulsions penetrate the leather and transform into water-in-oil emulsions, creating a hydrophobic layer with low surface tension that enhances water resistance.^54^ The water resistance test results of the leathers produced with different ratios of cationic lubricating emulsions in the study are listed in Table 7.

**Table 7:** Dynamic Water Absorption of Treated Leathers

samples	dynamic water absorption (%)	
	mean + SD	**SE**
control	125.2 ± 6.26	3.61
0.5% cationic emulsion	80.56 ± 4.43	2.56
1% cationic emulsion	80.65 ± 4.84	2.79
2% cationic emulsion	62.7 ± 4.08	2.35
3% cationic emulsion	60.3 ± 4.22	2.44
4% cationic emulsion	55.5 ± 4.16	2.40

Upon examining Table 7, it is clear that the application of cationic emulsions in varying concentrations has a substantial effect on the dynamic water absorption properties of the treated leathers. The control group, which did not receive any emulsion treatment, exhibited a significantly higher dynamic water absorption rate of 125.2 ± 6.26%. In contrast, as the emulsion concentration increased, a marked decrease in the water absorption was observed. Leathers treated with 0.5 and 1% cationic emulsions showed reductions in water absorption to 80.56 ± 4.43 and 80.65 ± 4.84%, respectively. Further increases in the emulsion concentration to 2, 3, and 4% led to progressively lower water absorption rates, with the 4% emulsion-treated leather achieving the lowest value at 55.5 ± 4.16%. This trend demonstrates that the hydrophobicity of the leather improves with the higher concentration of cationic emulsion, which supports the theory that emulsions containing hydrophilic and hydrophobic groups can create a water-repellent surface on the leather.^53^

### UV-Spectrophotometer Analysis Results

The results of dyestuff exhaustion in the dyeing float of leathers produced with various ratios of cationic lubricating emulsion are shown in Table 8.

**Table 8:** Dyestuff Exhaustion Results (%)

samples	dyestuff exhaustion (%)	
	mean + SD	**SE**
control	65.2 ± 3.26	1.88
0.5% cationic emulsion	75.54 ± 4.15	2.40
1% cationic emulsion	80.25 ± 4.82	2.78
2% cationic emulsion	82.8 ± 5.38	3.11
3% cationic emulsion	85.24 ± 5.97	3.44
4% cationic emulsion	88.98 ± 5.17	2.96

Upon analyzing Table 8, it is evident that the application of cationic emulsions at different concentrations leads to a significant increase in dyestuff exhaustion compared to the control group. In the control sample, where no emulsion was applied, the dyestuff exhaustion was recorded at 65.2 ± 3.26%. However, as the concentration of the cationic emulsion increased, there was a notable improvement in dye uptake. Specifically, the exhaustion increased to 75.54 ± 4.15% with the application of 0.5% emulsion, further rising to 80.25 ± 4.82, 82.8 ± 5.38, and 85.24 ± 5.97% and reaching its highest value at 88.98 ± 5.17% with 4% emulsion. In comparison to the control group, which had a dyestuff exhaustion of 65.2%, the highest emulsion concentration (4%) demonstrated an increase of over 23% in dye uptake.

This improvement highlights the significant role that cationic emulsions play in enhancing the dyeing process. The significant increase in dyestuff exhaustion with the use of cationic emulsions can be attributed to the improved interaction between the emulsion-treated leather and the anionic dyes used in the process. The cationic nature of the emulsion provides additional binding sites for the negatively charged anionic dyes, facilitating more efficient dye absorption.^55^ As the emulsion concentration increases, the availability of positively charged sites on the leather fibers increases, thereby facilitating a greater dye uptake. This effect becomes particularly pronounced at higher emulsion concentrations, where dyestuff exhaustion approaches 88.98%.

Notably, early applications of esterquats included their use as textile auxiliaries and dye leveling agents owing to their ability to stabilize dyeing baths and promote more uniform dye distribution across fabrics, ultimately improving the efficiency and consistency of the dyeing process. This same mechanism likely underpins their effectiveness in the current study, further demonstrating their versatility and utility in dyeing operations.^18^

## Conclusions

This study has demonstrated the significant potential of esterquat-based cationic fatliquoring emulsions as an innovative and eco-friendly solution for both the leather and textile industries. The application of these emulsions in both leather and textile treatments has demonstrated significant improvements in product quality while also addressing environmental concerns associated with traditional leather processing methods.

In leather applications, esterquat-containing emulsions were found to enhance critical physical properties, such as softness, flexibility, tear resistance, and water repellence. These improvements were most pronounced at higher concentrations of the emulsion, particularly at 4%, where a substantial increase in dyestuff exhaustion was also observed, improving resource utilization in the dyeing process. The emulsions exhibited stability under varying temperatures and acidic conditions, maintaining their effectiveness throughout the leather treatment process. Importantly, the antibacterial tests revealed that the emulsions provided a high level of protection against *S. aureus* and *E. coli*, with a 79.46% reduction in *S. aureus* growth at a 3% emulsion concentration, exceeding the threshold for effective antibacterial treatments.

In textile applications, the emulsions were especially effective on cotton fabrics, significantly enhancing softness even at lower concentrations, while polyester fabrics required higher doses for similar improvements. The antibacterial properties were also more pronounced on cotton, making the emulsions a viable alternative to conventional softeners, particularly in applications where both fabric softening and antibacterial functionality are required.

The findings indicate that the use of cationic esterquat-based emulsions not only elevates the quality of leather and textiles but also aligns with the leather industry’s shift toward cleaner production technologies. By reducing chemical waste and enhancing the efficiency of fatliquoring and dyeing processes, these emulsions present a sustainable alternative to conventional methods, addressing the industry’s need for eco-friendly solutions. This research lays the groundwork for further innovations in fatliquoring agents that promote both environmental responsibility and high-performance leather products.
